# Respiratory syncytial virus infection induces heterologous protection against SARS-CoV-2 through γδ T cell-mediated trained immunity and the activation of SARS-CoV-2–reactive mucosal T cells

**DOI:** 10.1128/jvi.01658-25

**Published:** 2026-03-18

**Authors:** Awadalkareem Adam, Wenzhe Wu, Madison C. Jones, Haiping Hao, Aditi, Parimal Samir, Xiaoyong Bao, Tian Wang

**Affiliations:** 1 Department of Microbiology & Immunology, University of Texas Medical Branch 547647, Galveston, Texas, USA; 2 Sealy Institute for Vaccine Sciences, University of Texas Medical Branch 559814, Galveston, Texas, USA; 3 Department of Pediatrics, University of Texas Medical Branch 12338, Galveston, Texas, USA; 4 Department of Biochemistry & Molecular Biology, University of Texas Medical Branch 198643, Galveston, Texas, USA; 5 Institute for Human Infections and Immunity, University of Texas Medical Branch 551582, Galveston, Texas, USA; 6 Department of Pathology, University of Texas Medical Branch 198642, Galveston, Texas, USA; Fred Hutchinson Cancer Center Vaccine and Infectious Disease Division, Seattle, Washington, USA

**Keywords:** RSV, trained immunity, SARS-CoV-2, γδ T cells

## Abstract

Respiratory viruses can infect hosts concurrently or sequentially, potentially influencing each other’s pathogenic trajectory. However, the underlying immune mechanisms governing these interactions remain poorly understood. In this study, we examined whether respiratory syncytial virus (RSV) infection modulates host susceptibility to subsequent SARS-CoV-2 infection using two murine models. We found that prior RSV infection conferred dose- and time-dependent heterologous protection against SARS-CoV-2. Transcriptomic and immunological analyses revealed that RSV activated lung antigen-presenting cells (APCs) and SARS-CoV-2–reactive mucosal T cells by day 9 post-infection, with responses waning by 1 month. RSV also promoted expansion of pulmonary γδ T cells and upregulation of their metabolic pathways. Notably, RSV-infected TCRδ⁻^/^⁻ mice, which lack γδ T cells, exhibited diminished SARS-CoV-2–reactive mucosal T cell responses, elevated viral loads, and exacerbated lung inflammation following SARS-CoV-2 challenge compared to wild-type controls. These findings suggest that RSV infection induces γδ T cell-mediated trained immunity and primes mucosal T cell responses, thereby providing heterologous protection against SARS-CoV-2.

**IMPORTANCE:**

The mechanisms by which prior respiratory viral infections confer heterologous protection remain largely undefined. In this study, we investigated whether respiratory syncytial virus (RSV) infection influences host susceptibility to subsequent SARS-CoV-2 infection in mice. We found that prior RSV exposure conferred dose- and time-dependent heterologous protection against SARS-CoV-2. Mechanistically, RSV infection induces γδ T cell-mediated trained immunity, enhances antigen-presenting cell activation, and promotes the generation of SARS-CoV-2–reactive mucosal T cells. Together, these immune responses contribute to cross-protective immunity against SARS-CoV-2. Our findings offer new insights into the immunological interplay between co-circulating respiratory viruses and SARS-CoV-2, with implications for future vaccine design and pandemic preparedness.

## INTRODUCTION

Severe acute respiratory syndrome coronavirus 2 (SARS-CoV-2), the etiological agent of coronavirus disease 2019 (COVID-19), has had a profound impact on global public health for over 4 years. Despite widespread vaccination and natural immunity, the virus continues to evolve and circulate within human populations. Alongside SARS-CoV-2, other respiratory viruses—including influenza virus, respiratory syncytial virus (RSV), human metapneumovirus (HMPV), and rhinovirus (RV)—are major contributors to acute respiratory illnesses, particularly in infants, the elderly, and immunocompromised individuals. These infections range from mild upper respiratory symptoms to severe lower respiratory tract diseases such as bronchiolitis and pneumonia and may exert long-term effects on host health beyond the resolution of acute illness. Respiratory viruses often co-circulate during winter and spring seasons and can infect hosts concurrently or sequentially. Such interactions may modulate host immunity, thereby influencing susceptibility to subsequent infections and altering disease severity (1). Notably, during the early phase of the COVID-19 pandemic, 20%–50% of healthy individuals with no prior exposure to SARS-CoV-2 exhibited cross-reactive CD4^+^ T cells against SARS-CoV-2 and endemic human coronaviruses (HCoVs) (2). In murine models, prior RSV infection has been shown to confer heterologous protection against unrelated respiratory viruses, including influenza and SARS-CoV-2 (3, 4). Nevertheless, the underlying mechanisms by which prior respiratory viral infections induce heterologous protection remain largely unknown.

Trained immunity, also termed innate immune memory, has been implicated in mediating host defense mechanisms following vaccination and microbial exposure. Epidemiological studies have demonstrated that live attenuated vaccines, such as Bacillus Calmette-Guerin (BCG) vaccine, Measles, mumps and rubella (MMR) vaccine, polio vaccine, and influenza vaccines, can confer nonspecific protection against unrelated infections through the induction of trained immunity (5–8). Animal model studies also showed that trained immunity induced by cytomegalovirus or bacterial infections can confer broad-spectrum protection against subsequent, unrelated microbial challenges (9–11). In this study, we investigated the effects of prior RSV infection on host susceptibility against subsequent SARS-CoV-2 challenge in two animal models. We found that RSV infection induces γδ T cell-mediated trained immunity, promotes the generation of SARS-CoV-2–reactive mucosal T cells, and thereby confers heterologous protection against subsequent SARS-CoV-2 infection.

## RESULTS

### Heterologous protection against subsequent SARS-CoV-2 infection induced by prior RSV exposure is both dose- and time-dependent

**Fig 1:**
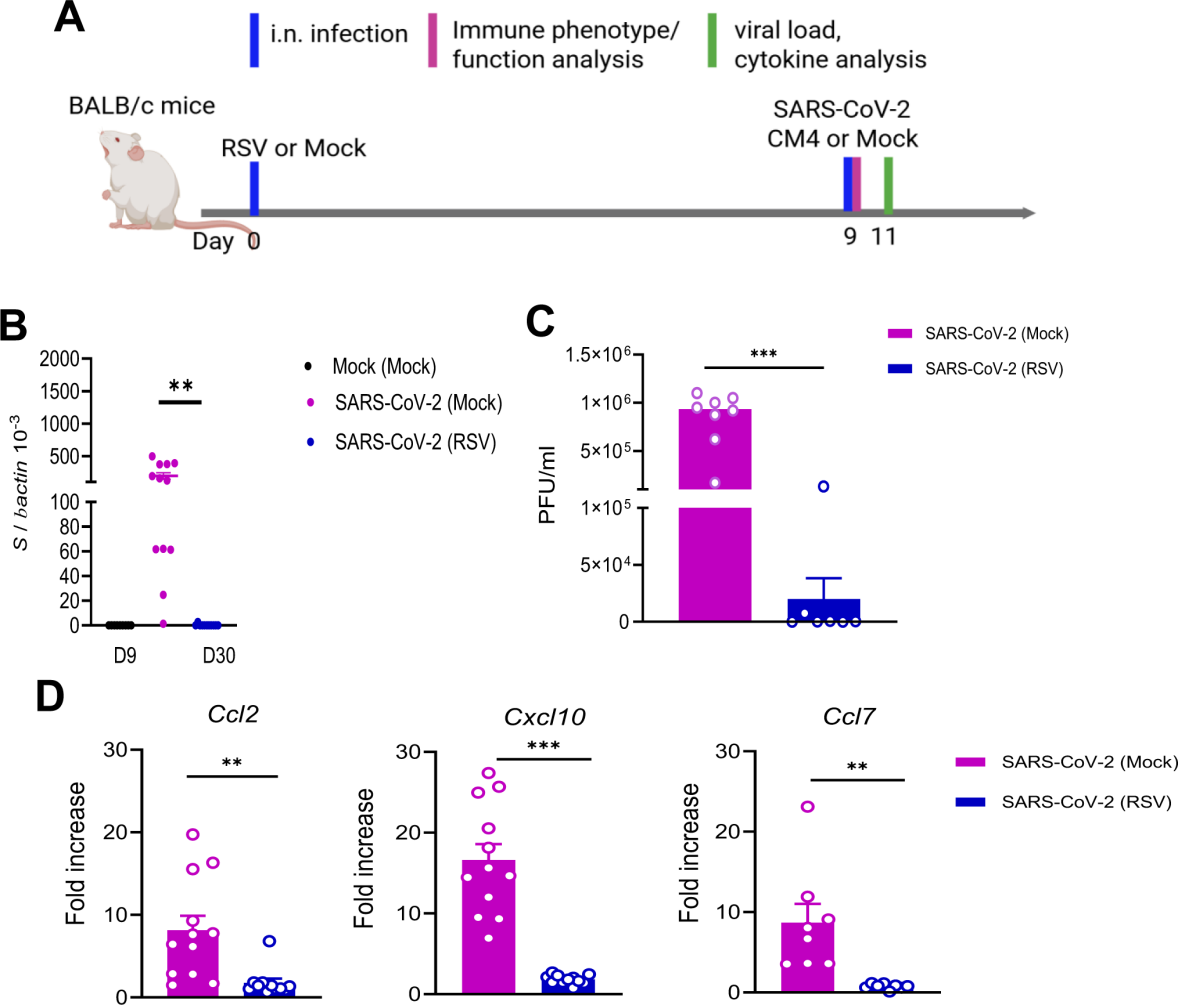
Prior RSV infection increased host resistance to subsequent SARS-CoV-2 infection in BALB/C mice. BALB/c mice were infected i.n. with RSV or PBS (mock), and on day 9, mice were challenged with mouse-adaptive SARS-CoV-2 strain CMA4. (**A**) Study design (created with BioRender.com). (**B–D**) Lung tissues were collected at day 2 post SARS-CoV-2 challenge. (**B and C**) Lung viral load measured by Q-PCR of spike (*S*) gene expression (**B**) and plaque assays (**C**). (**D**) Lung chemokine gene expression levels at day 2 post challenge determined by Q-PCR assay. Data are presented as fold increase compared to mock-infected mice. *n* = 9 to 12. **P < 0.01 or ***P < 0.001 SARS-CoV-2-infected mice with RSV prior infection (SARS-CoV-2 [RSV]) compared to SARS-CoV-2-infected mice with mock prior infection (SARS-CoV-2 [mock]). ^###^*P* < 0.001 compared to mock (mock) group.

**Fig 2:**
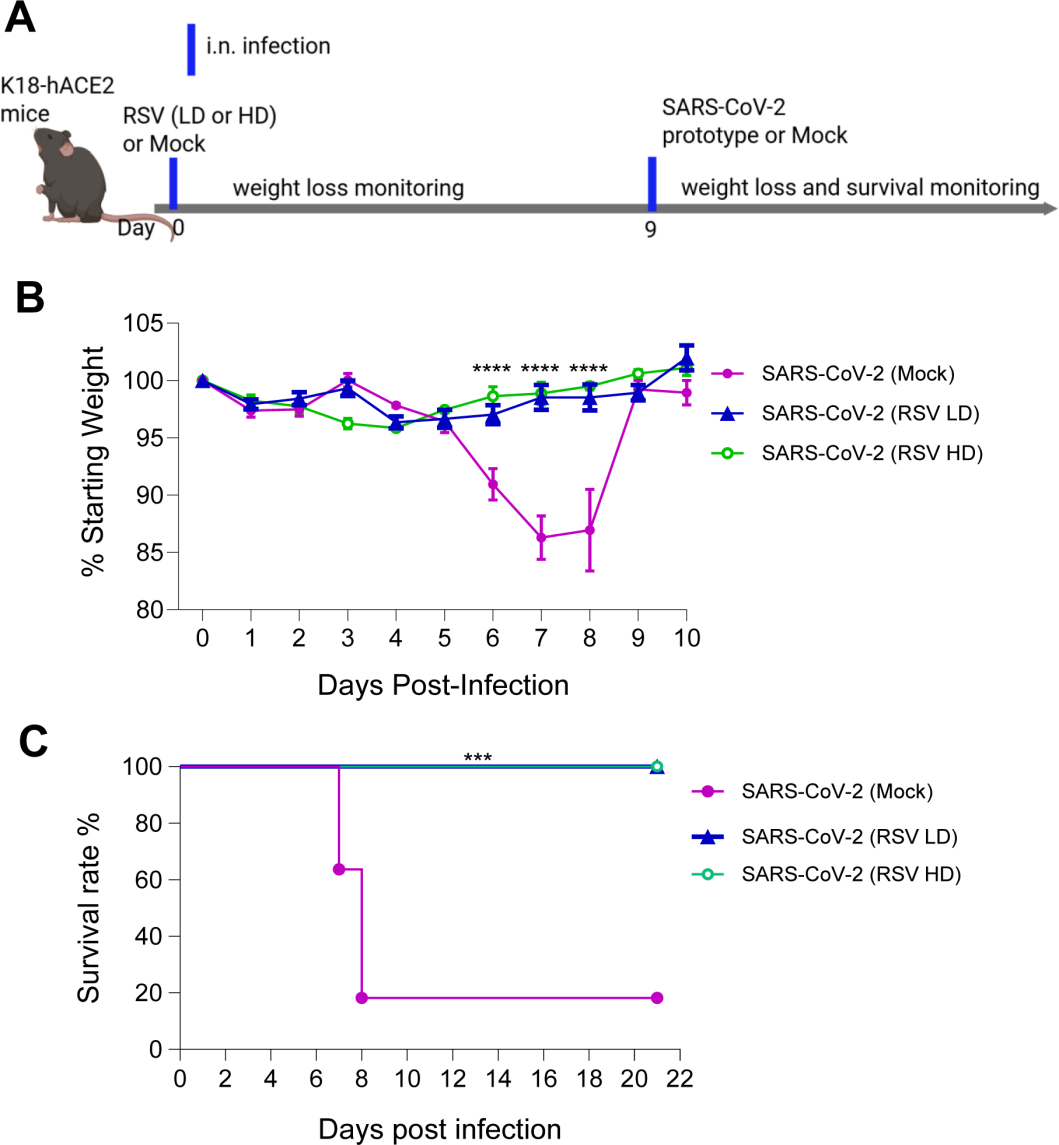
Prior RSV infection increased subsequent host survival from SARS-CoV-2 challenge in K18-hACE2 mice. K18-hACE2 mice were infected with a low (LD) or high dose (HD) of RSV or mock, and on day 9, mice were challenged with SARS-CoV-2 prototype strain. (**A**) Study design (created with BioRender.com). Mice were monitored daily for weight loss (**B**) or survival (**C**). Weight loss is indicated by percentage using the weight on the day of infection as 100%. ****P < 0.0001 or ***P < 0.001, (SARS-CoV-2 [RSV LD], *n* =13) or (SARS-CoV-2 [RSV HD], *n* = 6) compared to (SARS-CoV-2 [mock], *n* = 8).

To investigate the effects of RSV infection on protection against heterologous SARS-CoV-2 challenge, we initially employed the immunocompetent BALB/c mouse model. Mice were intranasally (i.n.) inoculated with 5 × 10⁶ PFU of the RSV A2 strain or PBS (mock control) and monitored daily for weight loss and morbidity. On day 9 post-RSV infection, mice were subsequently challenged i.n. with 1 × 10⁴ PFU of the mouse-adapted SARS-CoV-2 strain CMA4. (Fig. 1A). The mouse-adapted strain infects the lungs, causes inflammatory responses, and reaches the infection peak around day 2 (12, 13). To assess SARS-CoV-2- induced disease severity, we measured viral loads and chemokine levels in the lung 2 days after SARS-CoV-2 challenge. SARS-CoV-2-infected mice with prior RSV infection [SARS-CoV-2 (RSV)] showed markedly reduced viral loads in the lung as determined by Q-PCR and plaque assay (Fig. 1B and C). There were 4- to 11-fold lower levels of gene expression for chemokines, including *Ccl2*, *Cxcl10*, and *Ccl7* (Fig. 1D) compared to SARS-CoV-2-infected mice with prior mock infection [SARS-CoV-2 (Mock)]. K18-hACE2 transgenic mice express the hACE2 protein under the human keratin 18 (K18) promoter and are in C57BL/6 (B6) genetic background, which confers efficient transgene expression in airway epithelial cells. Acute SARS-CoV-2 infection in K18-hACE2 transgenic mice induces more pronounced weight loss, interstitial pneumonitis, encephalitis, and death (14–17). To further investigate how prior RSV infection influences host susceptibility to subsequent SARS-CoV-2 infection, K18-hACE2 mice were first infected with RSV at two different doses: a low dose (LD, 5 × 10⁶ PFU) and a high dose (HD, 1 × 10⁷ PFU). Infection by both doses of RSV resulted in weight loss in K18-hACE2 mice within a week of infection with a greater magnitude observed in the HD group (Fig. S1). On day 9 of the RSV infection, mice were challenged with 2 × 10^3^ PFU SARS-CoV-2 prototype strain to assess the impact of RSV pre-exposure (Fig. 2A). Mice with prior mock infection started to lose weight on day 6, and about 80% of them succumbed to SARS-CoV-2 infection within a 3-week interval. In contrast, none of the mice with prior LD or HD RSV infection displayed weight loss, and all survived subsequent SARS-CoV-2 infection (Fig. 2B and C).

**Fig 3:**
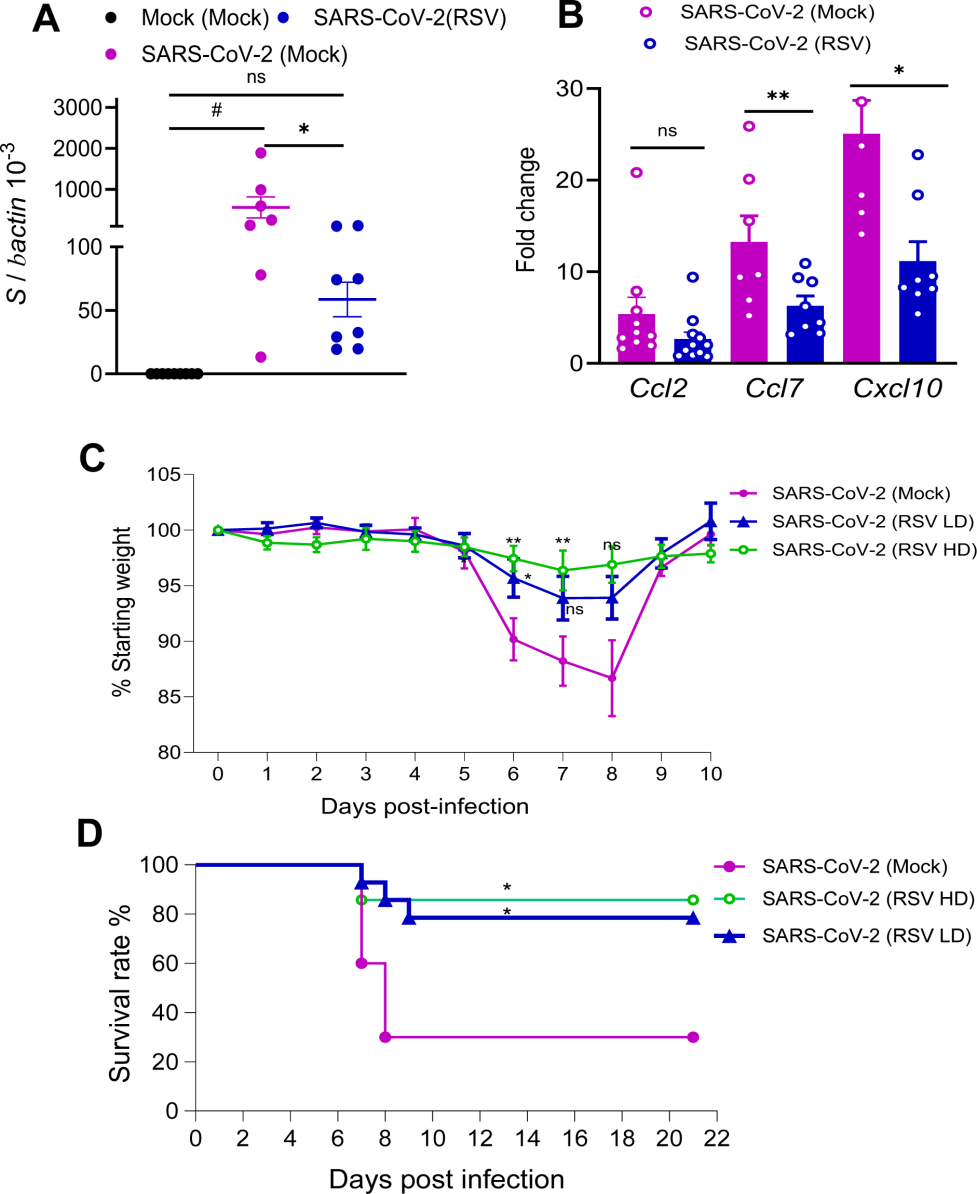
RSV-induced heterologous protection against SARS-CoV-2 declined at day 30 post RSV infection. (**A and B**) BALB/c mice were infected i.n. with RSV, and on day 30 post infection, mice were challenged with mouse-adaptive SARS-CoV-2 strain CMA4. Lung tissues were collected at day 2 post SARS-CoV-2 challenge. Lung viral loads (**A**) and chemokine gene expression levels (**B**) were measured by Q-PCR assay. Data are presented as fold increase compared to mock-infected. *n* = 7 to 8. (**C and D**) K18 hACE2 mice were infected with a low (LD) or high dose (HD) of RSV or mock, and on day 30 post RSV infection, mice were challenged with SARS-CoV-2 prototype strain. Mice were monitored daily for weight loss (**C**) and survival (**D**). Weight loss is indicated by percentage using the weight on the day of infection as 100%. *P < 0.05 or **P < 0.01 (SARS-CoV-2 [RSV LD], *n* = 14), (SARS-CoV-2 [RSV HD], *n* =6) compared to (SARS-CoV-2 [mock], *n* = 10). ^#^*P* < 0.05 compared to the mock (mock) group.

To determine the durability of RSV-induced heterologous protection, BALB/c mice were challenged i.n. with 1 × 10⁴ PFU of the SARS-CoV-2 CMA4 strain 30 days following RSV infection. At day 2 post-challenge, lung viral loads did not differ significantly between mice previously infected with RSV and those mock-infected (Fig. 3A). However, prior RSV-infected mice exhibited 1.5- to 3-fold lower levels of pulmonary gene expression of *Ccl7* and *Cxcl10* compared to mock-infected controls, while *Ccl2* levels remained comparable between the two groups (Fig. 3B). K18-hACE2 mice were challenged intranasally with 2 × 10³ PFU of the SARS-CoV-2 prototype strain on day 30 following either LD or HD RSV infection or mock treatment. All groups exhibited weight loss by day 6 post-SARS-CoV-2 challenge; however, mice with prior RSV infection (both LD and HD) experienced significantly less weight loss compared to mock-infected controls (Fig. 3C). On days 7 and 8 post-SARS-CoV-2 challenge, mice with prior HD RSV infection continued to exhibit reduced weight loss compared to the mock-infected group. In contrast, the difference in weight changes between the LD RSV group and mock controls was no longer statistically significant. Furthermore, mice with prior RSV infection exhibited enhanced survival following SARS-CoV-2 challenge, with survival rates of 78.5% in the LD RSV group and 85.7% in the HD RSV group, compared to only 30% survival in the mock-infected controls (Fig. 3D). Overall, these findings indicate that prior RSV infection reduces host susceptibility to subsequent SARS-CoV-2 challenge in both BALB/c and K18-hACE2 mouse models. The observed RSV-induced heterologous protection is both dose-dependent and time-sensitive.

### Transcriptomic and functional analysis reveals that RSV infection triggered the activation of antigen presenting cells and SARS-CoV-2-reactive mucosal T cell responses

**Fig 4:**
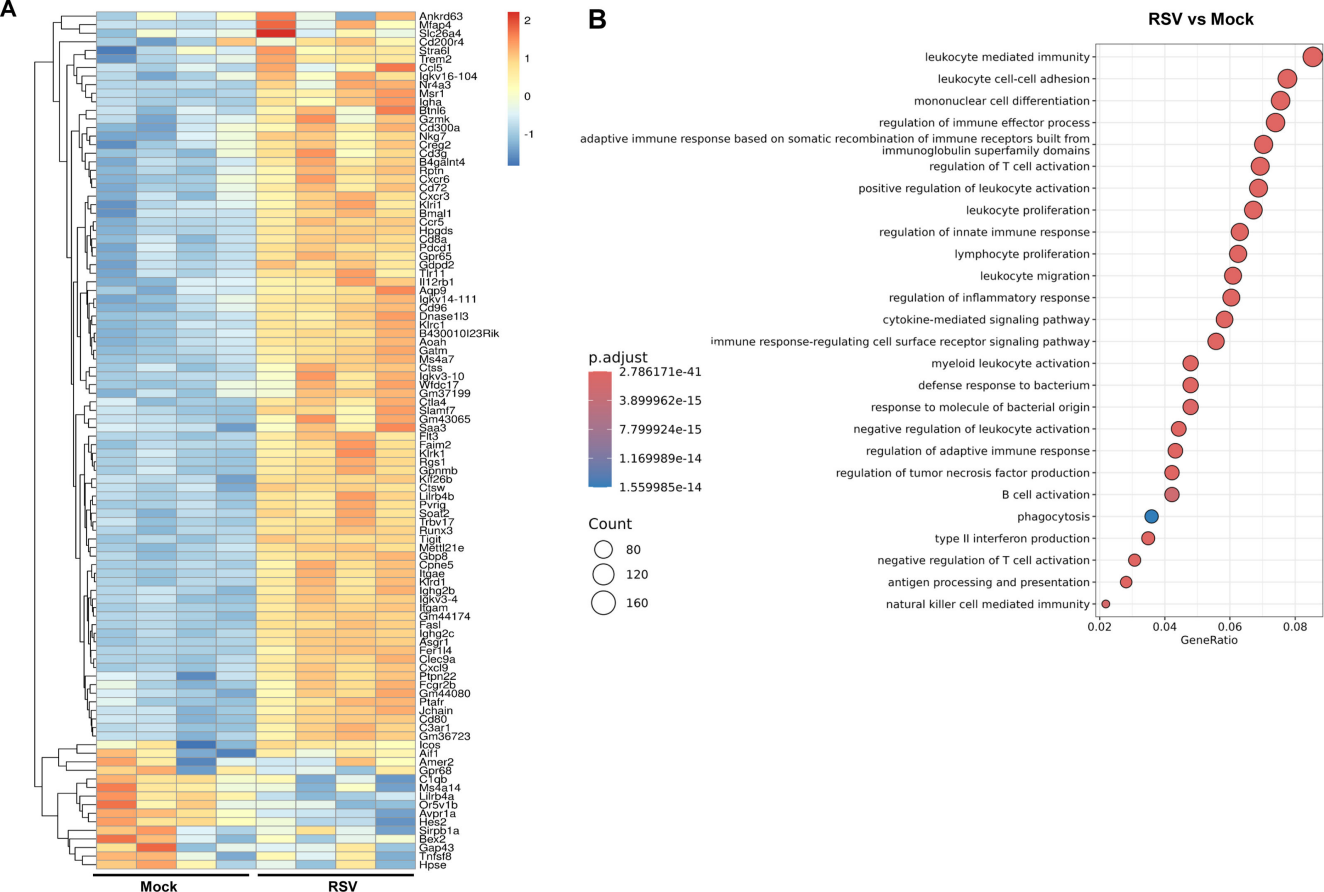
Transcriptome analysis of lung samples of RSV-infected mice. B6 mice were infected i.n. with RSV or mock, and on day 9 post-infection, lung tissue RNA was isolated for RNAseq analysis. (**A**) Heatmap of the top 100 differentially expressed genes in RSV-infected lung tissues compared to the mock controls at day 9. (**B**) A simplified Gene Ontology (GO) enrichment dot plot showing the most significant signaling pathways induced by RSV infection compared to mock infection.

To investigate the immune mechanisms underlying RSV-induced heterologous protection, B6 mice were infected with RSV or mock-treated. On day 9 post-RSV infection, mice were challenged with 1 × 10⁴ PFU of SARS-CoV-2 CMA4 or mock. Lung tissues were harvested at day 9 post-RSV infection and day 2 post-SARS-CoV-2 challenge for transcriptomic analysis via RNA sequencing. RSV infection induced the top 100 differentially expressed genes, which were enriched in multiple immune-related pathways, including “leukocyte cell-cell adhesion,” “regulation of T cell and lymphocyte activation,” “lymphocyte proliferation,” “innate immune response regulation,” “antigen receptor-mediated signaling,” “type II interferon signaling,” “innate immunity activation,” “leukocyte activation,” and “antigen processing” (Fig. 4A and B; Fig. S2A; Table S1). Gene set enrichment analysis (GSEA) of the transcriptomics data set revealed significant upregulation of immune-related pathways in the RSV-infected group compared to the mock group (Fig. S2B through D). Notably, pathways involved in “lymphocyte-mediated immunity,” “leukocyte-mediated immunity,” and “adaptive immune responses” showed marked enrichment in the RSV group. The “lymphocyte-mediated immunity” pathway exhibited a strong positive enrichment score (NES = 2.48), indicating elevated expression of genes associated with lymphocyte activation and effector functions in RSV-infected samples. Similarly, the “leukocyte-mediated immunity” pathway was significantly enriched (NES = 2.40), suggesting enhanced recruitment and activation of leukocytes in response to RSV infection. The “adaptive immune response*”* pathway was also prominently upregulated (NES = 2.37), reflecting the activation of antigen-specific immune mechanisms, including T and B cell responses. These findings were further supported by corresponding heatmaps, which display higher expression levels of genes within these pathways in the RSV group compared to mock. Interestingly, a similar set of upregulated genes and associated immune signaling pathways was observed in SARS-CoV-2–infected mice with prior RSV exposure, compared to mock-infected controls (Fig. S3). Collectively, these findings suggest that RSV-induced heterologous protection against SARS-CoV-2 is mediated through the activation of APCs and T cell responses.

**Fig 5:**
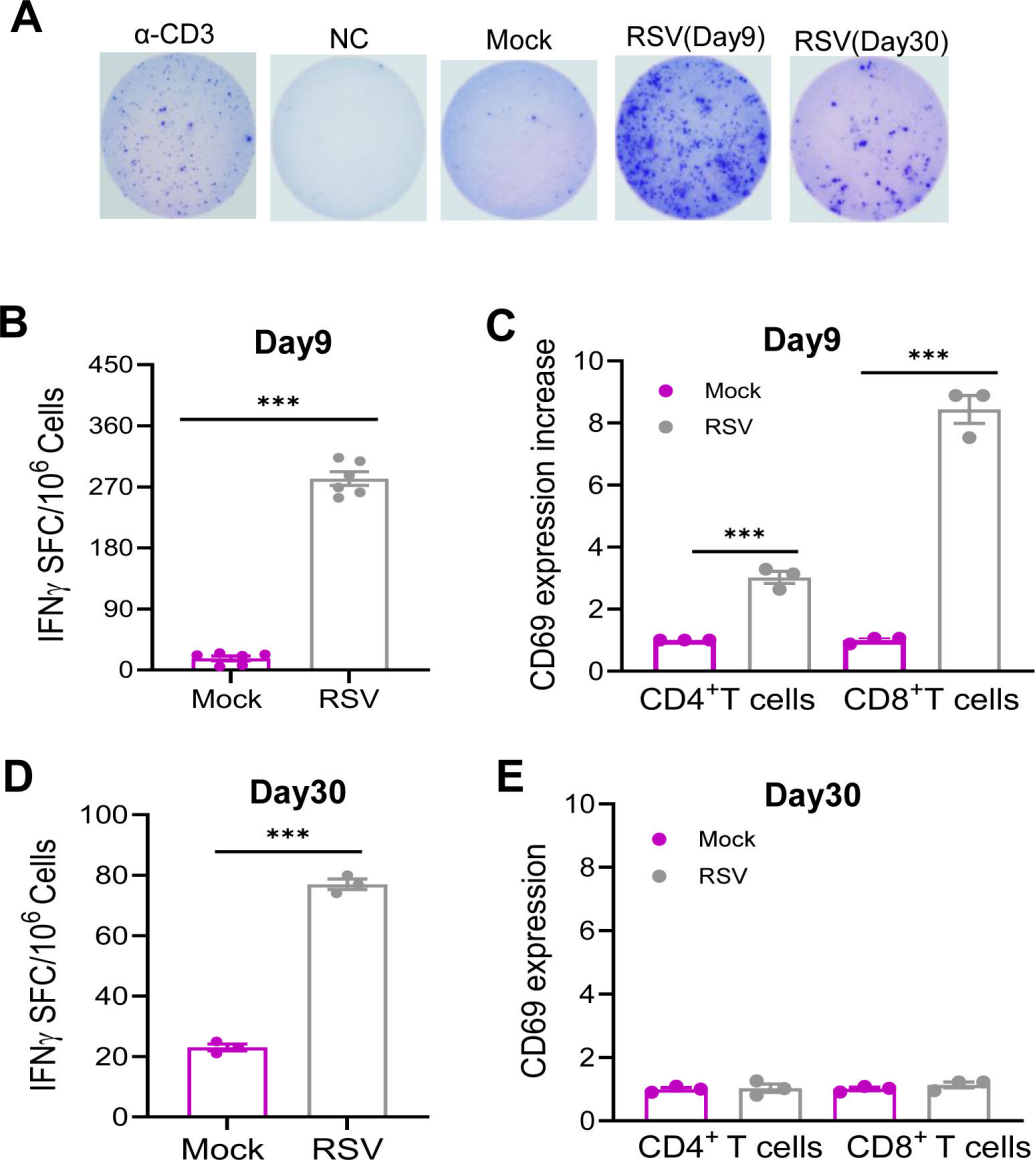
RSV induced SARS-CoV-2-reactive mucosal T cell responses. BALB/c mice were infected i.n. with RSV or PBS (mock) and on day 9 (**B and C**) or day 30 (**D and E**) post infection. Lung leukocytes were stimulated with SARS-CoV-2 S & N peptides, α-CD3, or blank for 36 h. (**A**) Images of wells from T cell culture. (**B and D**) Spot forming cells (SFC) were measured by IFN-γ ELISpot. Data are shown as # of SFC per 10^6^ lung leukocytes. *n* = 3 to 6. (**C and E**) CD69 expression on lung T cell subsets. Cells were gated in total lung leukocytes. Data are presented as fold increase compared to the mock-infected. ***P < 0.001 RSV-infected compared to mock-infected.

**Fig 6:**
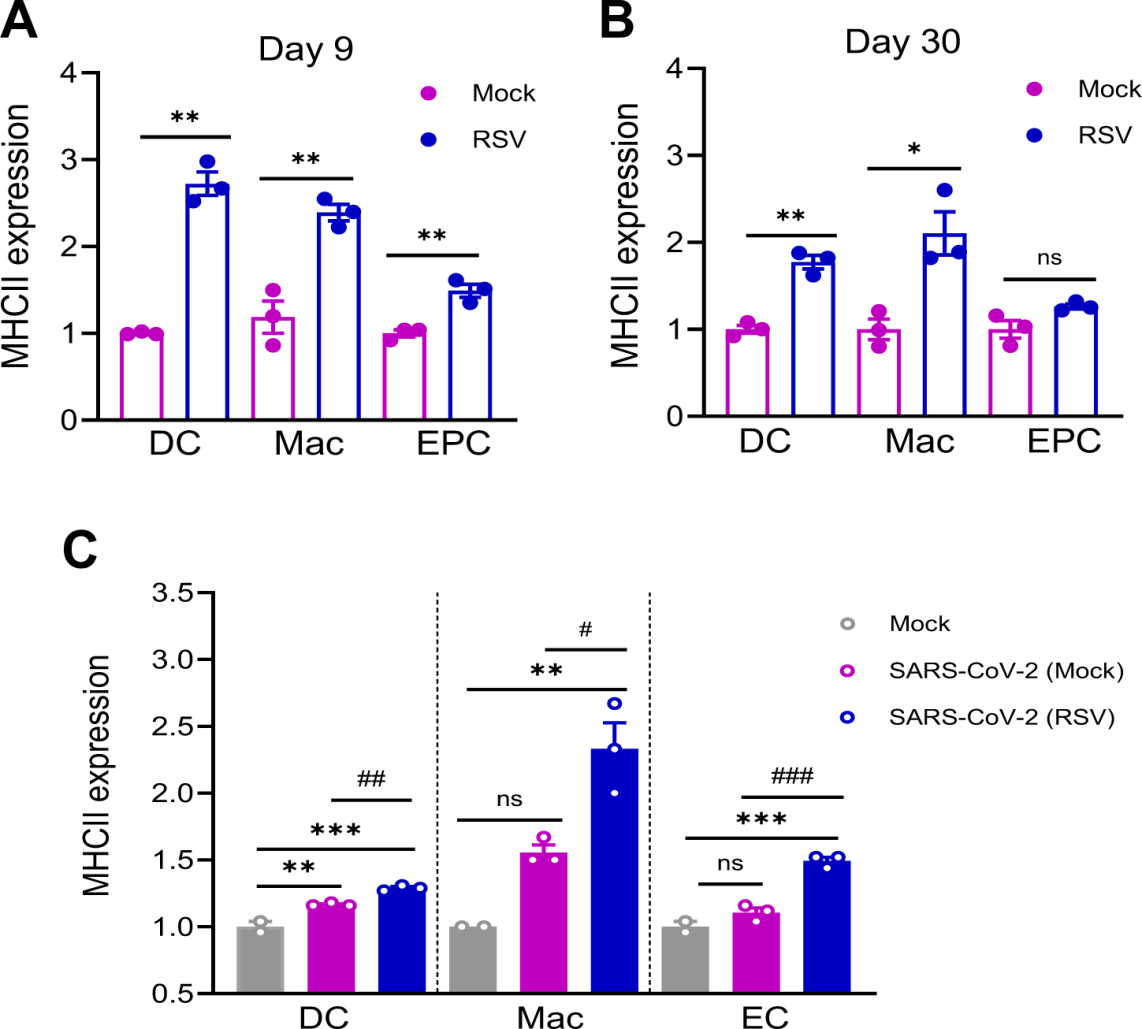
RSV-induced the activation of antigen presentation cells (APCs) in the lung. (**A and B**) BALB/c mice were infected i.n. with RSV or PBS (mock). On day 9 (**A**) and day 30 (**B**) post infection, lung APCs were stained for CD11c (DC, dendritic cells), F4/80 (macrophages, Mac), CD326 (epithelial cells, EC), and MHC class II. (**C**) B6 mice were infected i.n. with RSV or mock, and on day 30 post infection, mice were challenged with mouse-adaptive SARS-CoV-2 strain CMA4. Lung leukocytes were isolated at day 2 post SARS-CoV-2 challenge and stained for APC markers (CD11C, F4/80, and CD326) and MHC II. Cells were gated in total lung leukocytes. Data are presented as fold increase compared to mock-infected. *n* = 2 to 3. Data are one representative of two similar experiments. ***P < 0.001, **P < 0.01, or *P < 0.05 compared to mock group. ^###^*P* < 0.001, ^##^*P* < 0.01, or ^#^*P* < 0.05 compared to SARS-CoV-2 (mock) group.

To validate the RNA-seq findings, we assessed lung T cell responses in RSV-infected mice. Lung leukocytes were isolated on day 9 and day 30 post-RSV infection. As shown in Fig. 5A and B, ELISPOT assays revealed that RSV infection elicited over a 16-fold increase in SARS-CoV-2 spike (S) protein-specific T cell responses compared to mock-infected controls. Additionally, RSV-infected mice exhibited a threefold and eightfold increase in CD69 expression—an early T cell activation marker—on lung CD4^+^ and CD8^+^ T cells, respectively, relative to mock-infected mice (Fig. 5C). SARS-CoV-2–specific IgG and IgA antibodies in the sera and bronchoalveolar lavage (BAL) fluid of RSV-infected BALB/c and B6 mice were barely detectable at day 9 post-infection (Fig. S4A through D). By day 30, SARS-CoV-2–reactive lung T cell responses had diminished though they remained approximately threefold higher than those in mock-infected controls (Fig. 5D). CD69 expression on lung CD4^+^ and CD8^+^ T cells was reduced, with no significant differences observed between RSV- and mock-infected groups, indicating decreased T cell activation at this later time point (Fig. 5E). Furthermore, SARS-CoV-2–specific IgG antibodies remained undetectable in the sera of RSV-infected mice at day 30 (Fig. S4E). These findings suggest that RSV-induced pulmonary T cell responses reactive to SARS-CoV-2 peak at day 9 but wane by day 30. Given that transcriptomic analysis indicated the induction of APCs and innate immune signaling pathways following RSV infection, we next validated these findings by assessing lung APC activation. Specifically, we measured MHC class II expression on lung macrophages (F4/80^+^), dendritic cells (DCs, CD11c^+^), and epithelial cells (CD326^+^) in RSV-infected BALB/c mice. At day 9 post-infection, MHC class II expression levels were elevated by 1.5- to 2.7-fold on macrophages, DCs, and epithelial cells compared to mock-infected controls (Fig. 6A). By day 30, MHC class II induction was diminished on DCs and epithelial cells but remained unchanged on macrophages (Fig. 6B). Furthermore, mice challenged with a mouse-adapted SARS-CoV-2 strain at day 9 post-RSV infection exhibited a 1.5-fold increase in MHC class II expression on day 2 compared to those with mock prior infection, suggesting that RSV infection may induce a trained immunity phenotype (Fig. 6C).

### RSV confers heterologous protection against subsequent SARS-CoV-2 infection, in part, through modulation of γδ T cell function

**Fig 7:**
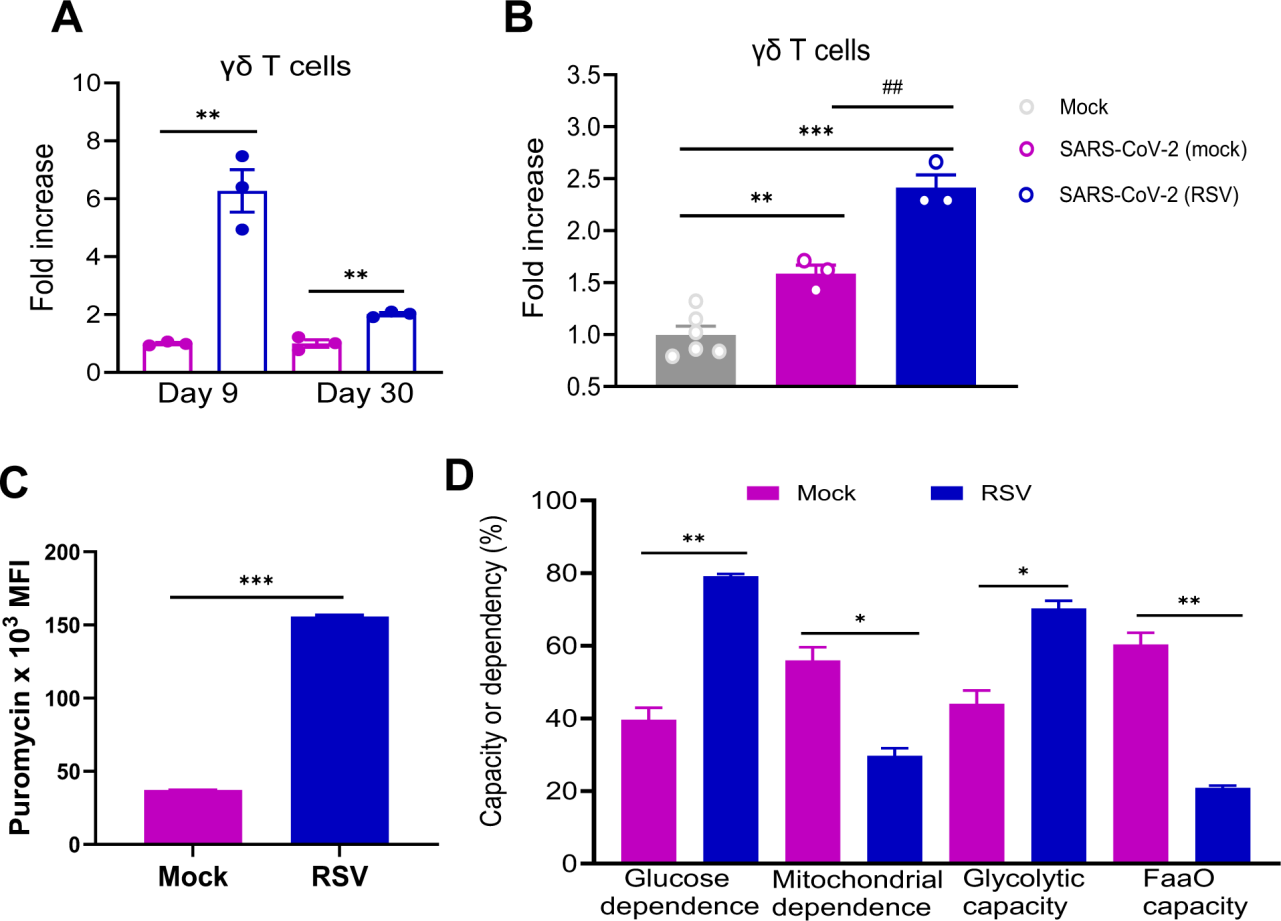
RSV-induced trained γδ T cell responses. (**A**) BALB/c mice were infected i.n. with RSV or PBS (mock). On day 9 and day 30 post-infection, lung leukocytes were stained for CD3 and TCRγδ. Cells were gated in total lung leukocytes for percent positive and data are presented as fold increase compared to mock-infected. (**B**) B6 mice were infected i.n. with RSV or mock, and on day 30 post infection, mice were challenged with mouse-adaptive SARS-CoV-2 strain CMA4. Lung leukocytes were isolated at day 2 post SARS-CoV-2 challenge and stained for CD3 and TCRγδ. Cells were gated in total lung leukocytes for percent positive, and data are presented as fold increase compared to mock-infected. *n* = 3 to 6. (**C and D**) Lung leukocytes were isolated from RSV or mock-infected BALB/c mice at day 9 pi. Metabolic parameters by modified SCENITH (https://www.scenith.com), calculated as described in reference 18. (**C**) puromycin incorporation, (**D**) glycolytic capacity, fatty acid oxidation/amino acid oxidation (FAO/AAO) capacity, mitochondrial dependence, and glucose dependence. All parameters were measured by flow cytometry. Data are one representative of three similar experiments. ***P < 0.001, **P < 0.01, or *P < 0.05 compared to mock group. ^##^*P* < 0.01 compared to SARS-CoV-2 (mock) group.

Cross-talk between γδ T cells and APCs is known to play a critical role in APC activation. In RSV-infected BALB/c mice, lung γδ T cells expanded more than 6-fold by day 9 post-infection and declined to a 2-fold increase by day 30, coinciding with the waning RSV-induced protection against SARS-CoV-2 (Fig. 7A). Moreover, SARS-CoV-2 infection in B6 mice elicited a 1.6-fold greater expansion of γδ T cells in animals previously infected with RSV compared to mock-infected controls (Fig. 7B). These findings suggest that RSV infection may induce a trained γδ T cell response.

To investigate the functional metabolic profile of lung γδ T cells in RSV-infected mice, we employed the SCENITH method—a flow cytometry-based technique that uses puromycin incorporation as a proxy for protein synthesis and cellular ATP usage. RSV infection led to a substantial increase in protein synthesis levels, as indicated by elevated puromycin mean fluorescence intensity (MFI) in γδ T cells (Fig. 7C). Using metabolic inhibitors, we further assessed fatty acid/amino acid oxidation capacity, glycolytic capacity, mitochondrial dependence, and glucose dependence. Lung γδ T cells from RSV-infected mice exhibited enhanced glycolytic capacity and reduced reliance on mitochondrial energy metabolism (Fig. 7D; Fig. S5A and B). Additionally, these cells showed decreased fatty acid/amino acid oxidation capacity alongside increased glucose dependence, highlighting a metabolic shift toward glycolysis following RSV infection.

**Fig 8:**
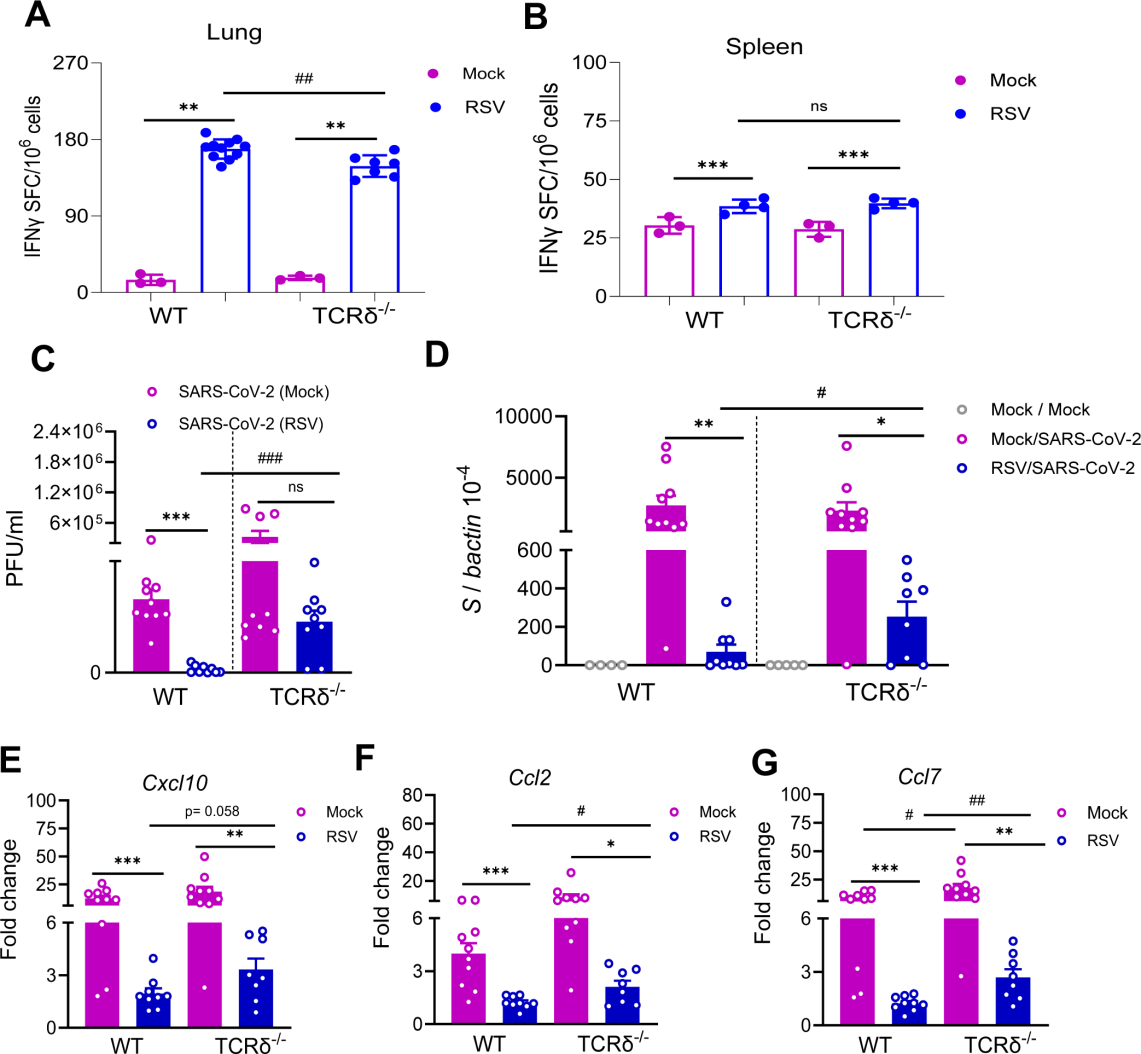
RSV-induced γδ T cell activation contributed to heterologous protection against subsequent SARS-CoV-2 challenge. WT B6 and TCRδ^−/−^ mice were infected i.n. with 5 × 10^6^ PFU RSV A2 or mock. (**A and B**) Lung leukocytes (**A**) or splenocytes (**B**) were stimulated with SARS-CoV-2 S & N peptides, or blank for 24 to 36 h. Spot forming cells (SFC) were measured by IFN-γ ELISpot. Data are shown as # of SFC per 10^6^ lung leukocytes. *n* = 3 to 11. (**C–G**) At day 9 of RSV infection, all mice were challenged with 1 × 10^4^ PFU mouse-adapted SARS-CoV-2 strain CMA4. Two days after viral challenge, lung tissues were collected. SARS-CoV-2 viral titers in lung tissues were measured by plaque (**C**) and Q-PCR (**D**) assays. (**E–G**) Measurement of chemokine gene expression levels in lung tissues by Q-PCR assays at day 2 post-infection. Data are presented as the fold increase compared to naïve mice (means ± SEM). *n* = 8 to 10. ***P < 0.001, **P < 0.01, or *P < 0.05 compared to mock group. ^###^*P* < 0.001, ^##^*P* < 0.01, or ^#^*P* < 0.05 compared to WT group.

To assess the contribution of γδ T cells to the induction of SARS-CoV-2–reactive T cell responses, WT B6 and TCRδ^−/−^ mice were intranasally infected with 5 × 10⁶ PFU of RSV A2 or mock-treated. By day 9 post-infection, both RSV-infected WT and TCRδ^−/−^ mice exhibited Th1-skewed SARS-CoV-2–specific immune responses in the lung compared to their respective mock-infected controls. Notably, WT mice demonstrated over a onefold higher SARS-CoV-2–reactive T cell response in the lung relative to TCRδ^−/−^ mice, suggesting that RSV-induced pulmonary T cell responses are partially dependent on γδ T cells (Fig. 8A). SARS-CoV-2–specific IgA^+^ B cells were scarcely detectable in the lungs of either group (Fig. S5C). In the spleen, no significant differences were observed between WT and TCRδ^−/−^ mice although both groups exhibited low levels of SARS-CoV-2–specific T cell responses (Fig. 8B). Lastly, to determine whether γδ T cells contribute to RSV-induced heterologous protection, WT B6 and TCRδ^−/−^ mice were intranasally infected with 5 × 10⁶ PFU of RSV A2 or mock-treated. Mice were monitored daily for weight loss and morbidity. TCRδ^−/−^ mice exhibited greater weight loss within 6 days post-RSV infection compared to WT mice (Fig. S5D). On day 9 post-infection, all mice were challenged with 1 × 10⁴ PFU of mouse-adapted SARS-CoV-2 strain CMA4. WT mice with prior RSV infection showed significantly lower levels of infectious particles and viral RNA compared to WT mice with prior mock infection (Fig. 8C and D). Although TCRδ^−/−^ mice with prior RSV infection also demonstrated reduced viral RNA levels relative to their mock-infected counterparts, they exhibited significantly higher levels of infectious particles and viral RNA than WT mice with prior RSV infection. Additionally, TCRδ^−/−^ mice with prior RSV infection displayed elevated chemokine levels compared to WT mice, indicating increased lung pathology in the absence of γδ T cells (Fig. 8E through G). Collectively, these findings suggest that RSV reprograms the metabolic profile of lung γδ T cells, which, in turn, contribute to the generation of SARS-CoV-2–reactive T cells and confer heterologous protection against SARS-CoV-2 challenge.

## DISCUSSION

Prior vaccination or infection with other respiratory viruses can influence the course of SARS-CoV-2 infection. Notably, influenza and BCG vaccines have been reported to confer protection against SARS-CoV-2 through the induction of trained immunity (19–22). Additionally, one recent report demonstrated that prior RSV infections provide heterologous protection against SARS-CoV-2 infection (4). However, the underlying mechanisms remain largely unclear. Trained immunity refers to a long-term enhancement in the responsiveness of innate immune cells, driven by metabolic, epigenetic, and transcriptional reprogramming (23, 24). Several innate immune cell types—including monocytes, macrophages, neutrophils, DCs, and natural killer cells—have been implicated in this process (23, 24). More recently, γδ T cells have emerged as key contributors to trained immunity, particularly in response to MMR vaccination. Here, we used two animal models to demonstrate that prior RSV infection induces heterologous protection against subsequent SARS-CoV-2 challenge via γδ T cell-mediated trained immunity. Although γδ T cells constitute a minority of CD3^+^ T cells in lymphoid tissues and peripheral blood, they are highly enriched at epithelial and mucosal sites. These cells are not restricted by MHC and instead express innate-like receptors such as toll-like receptors (TLRs) and NKG2D, enabling them to play a pivotal role in innate immune responses (18). Clinical and experimental evidence suggests that γδ T cells contribute to host defense against SARS-CoV-2 infection (25–27). We found that TCRδ^−/−^ mice experienced greater weight loss within 6 days following RSV infection compared with RSV-infected WT mice. RSV infection induced γδ T cell expansion and altered their cellular metabolic pathways. In addition, TCRδ^−/−^ mice with prior RSV infection exhibited elevated chemokine levels and higher viral loads than WT mice with prior RSV infection. Together, these findings indicate that γδ T cells play a critical role in protective immunity against RSV. Notably, RSV-reprogrammed γδ T cells also contribute to host protection against SARS-CoV-2 challenge. To further elucidate the mechanisms underlying γδ T cell-mediated trained immunity in heterologous protection, future studies will focus on molecular and epigenetic changes using integrated ATACseq and RNAseq at single cell resolution to link chromatin accessibility with transcriptional programs in individual immune cell types, including γδ T cells. In addition, ChIPseq analysis will be used to assess histone modifications, including increased H3K4me3 and H3K27ac levels. As trained immunity is closely associated with metabolic rewiring (including glycolysis, the TCA cycle, and the mevalonate pathway), based on our findings here, we speculate that treatment with 2-DG would inhibit the trained immunity program induced by RSV in γδ T cells and diminish heterologous protection against SARS-CoV-2 challenge.

γδ T cells are known to interact with APCs, promoting their maturation, enhancing T cell priming, and supporting memory T cell development during microbial infections (28–30). In this study, we observed heightened lung APC responses—including DCs, macrophages, and epithelial cells—following SARS-CoV-2 challenge in mice previously infected with RSV. Notably, RSV-infected TCRδ^−/−^ mice exhibited reduced frequencies of SARS-CoV-2-reactive T cells, suggesting that RSV-induced γδ T cells contribute to APC activation and subsequent T cell priming. Combined together, our results suggest that RSV reprograms the metabolic profile of lung γδ T cells to trigger the activation of these cells, which then promote APC maturation via crosstalk and SARS-CoV-2–reactive T cell priming and confer heterologous protection against SARS-CoV-2 challenge.

In addition to their role in cellular immunity, γδ T cells also mediate humoral responses by facilitating CD19^+^ B cell activation and immunoglobulin production (31, 32). Expansion of γδ T cells has been associated with elevated anti-SARS-CoV immunoglobulin G titers (33). However, in our study, RSV infection elicited minimal SARS-CoV-2-reactive B cell responses and low antibody titers in both peripheral and mucosal compartments. These findings suggest that RSV-induced γδ T cell expansion does not drive SARS-CoV-2-specific antibody production. Interestingly, TCRδ^−/−^ mice with prior RSV infection displayed reduced—but not entirely abolished—heterologous protection against SARS-CoV-2, along with diminished SARS-CoV-2-reactive mucosal T cells. We also found that lung APCs, including DCs, macrophages, and epithelial cells were activated post-RSV infection. Lung APCs in mice with prior RSV infection exhibited a greater upregulation of MHCII upon SARS-CoV-2 challenge compared to those with prior mock infection. In addition to cross-talk with γδ T cells, lung APCs may also be directly activated by RSV infection independent of γδ T cell signaling. Future studies will investigate this possibility. Overall, our findings suggest that γδ T cell-independent mechanisms—likely involving RSV-induced trained lung APCs—also contribute to the induction of SARS-CoV-2-reactive T cells and confer partial protection against viral challenge.

Two animal models—BALB/c mice and K18-hACE2 transgenic mice—were employed to investigate RSV-induced heterologous protection against SARS-CoV-2. Given that K18-hACE2 mice are generated on a B6 background, additional immunological analyses were conducted in both BALB/c and B6 mice to account for strain-specific immune responses. Our findings demonstrated that RSV infection induced SARS-CoV-2-reactive T cells in both models; however, these responses declined over time. Similarly, RSV-induced trained immunity, including activation of lung APCs and expansion of γδ T cells, diminished by one month post-infection. These observations suggest that RSV-induced trained immunity is transient and closely linked to its heterologous protective effects. Utilizing both mouse models enabled us to compare viral behavior in a natural mouse-adapted system vs a humanized ACE2-expressing model. This approach provided broader insights into RSV-induced trained immunity, especially given the distinct genetic and immunological backgrounds of the two strains. Overall, our study provides understanding of the interplay between co-circulating respiratory viruses and SARS-CoV-2, shedding light on host immune dynamics and viral pathogenesis. Importantly, trained immunity may serve as a promising strategy to enhance vaccine efficacy and bolster both specific and non-specific immune defenses. Investigating the influence of recent infections on SARS-CoV-2 susceptibility and vaccine performance could inform future immunization strategies and pandemic preparedness.

## MATERIALS AND METHODS  

### Viruses

SARS-CoV-2 USA-WA1/2020 strain was obtained from the World Reference Center for Emerging Viruses and Arboviruses (WRCEVA) at the University of Texas Medical Branch (UTMB) and was amplified twice in Vero E6 cells. Mouse-adapted SARS-CoV-2 strain CMA4 viral stocks were provided by Dr. Xuping Xie at UTMB. The generation of SARS-CoV-2 strain CMA4 was described previously (13). RSV long strain was grown in HEp-2 cells and purified by sucrose gradient as described previously (34).

### Mouse infection and tissue collection

Seven- to 8-week-old BALB/c and C57BL/(B)6 mice were purchased from Jackson Laboratory. γδ T cell-deficient mice (TCRδ^−/−^, stock #002120, Jackson Laboratory) and K18 hACE2 mice (stock #034860, Jackson Laboratory) were both on B6 background and were bred at the UTMB animal facility. Age- and sex-matched male and female mice were used in this study. Mice were infected intranasally (i.n) with 5 × 10^6^ or 1 × 10^7^ plaque-forming units (PFU) of RSV. At day 9 or day 30 post RSV infection, mice were challenged with 1 × 10^4^ PFU of the SARS-CoV-2 CMA4 strain or 2 × 10^3^ PFU of the SARS-CoV-2 USA-WA1/2020 strain. Infected mice were monitored twice daily for morbidity and mortality. In some experiments, infected mice were euthanized for tissue collection. The right superior and inferior lobes of lung tissues were then collected in Trizol for RNA extraction and in DMEM for viral titration by plaque assay, respectively (13).

### Quantitative PCR

Lung tissues were resuspended in TRIzol for RNA extraction according to the manufacturer’s instructions (Thermo Fisher). Complementary (c) DNA was synthesized by using a qScript cDNA synthesis kit (Bio-Rad, Hercules, CA). The sequences of the Q-PCR primer sets for mouse cytokines, chemokines, SARS-CoV-2 *S* gene, and PCR reaction conditions were described previously (35–37). The PCR assay was performed in the CFX96 real-time PCR system (Bio-Rad). Gene expression was calculated using the formula 2^−[*C*^*t*^(target gene)-*C*^*t*^(β-actin)]^ as described before (38).

### Plaque assay

Vero E6 cells were seeded in 6-well plates and incubated at 37°C. Ten-fold serially diluted lung tissue homogenates were used to infect cells at 37°C for 1 h. After the incubation, cells were overlaid with 2X-MEM (Thermo Fisher) with 8% FBS and 1.6% agarose (VWR). After 48 h incubation, plates were stained with 0.05% neutral red (Sigma-Aldrich), and plaques were counted to calculate virus titers expressed as PFU/mL.

### Antibody ELISA

ELISA plates (Corning, USA) were coated with 100 ng/well recombinant SARS-CoV-2 RBD protein (RayBiotech) for overnight at 4°C. The plates were washed twice with PBS containing 0.05% Tween-20 (PBS-T) and then blocked with 8% FBS for 1.5 h. Sera were diluted 1:100 in blocking buffer. Bronchoalveolar lavage (BAL) fluid was collected by making an incision in the trachea and lavaging the lung twice with 1 mL PBS. Following centrifugation, supernatant was collected to measure antibody titers. BAL fluid was diluted 1:3 in blocking buffer. Samples were added for 1 h at 37°C. Plates were washed five times with PBS-T. Goat anti-mouse IgG (Southern Biotech) coupled to horseradish peroxidase (HRP) or alkaline phosphatase. For IgA measurement, goat anti-mouse IgA (Southern Biotech) coupled to HRP was added at a 1:2,000 dilutions for 1 h at 37°C. The was followed by adding TMB (3, 3, 5, 5′- tetramethylbenzidine) peroxidase substrate (Thermo Fisher Scientific) for about 5 min. The reactions were stopped by 1M sulfuric acid, and the intensity was read at an absorbance of 450 nm.

### Flow cytometry

Lung leukocytes were stained with antibodies for CD3, TCRγδ, CD4, CD8, CD69, F4/80, CD11c, CD326, and MHC II (all antibodies were purchased from ThermoFisher). After staining, the cells were fixed in 1% paraformaldehyde and acquired by a C6 flow cytometer instrument (BD Biosciences). Dead cells were excluded based on forward and side light scatter. Data were analyzed with a CFlow Plus flow cytometer (BD Biosciences).

### IFN-γ ELISPOT

Millipore ELISPOT plates (Millipore Ltd) were coated with mouse anti-IFN-γ capture Ab at 1:100 dilution (Cellular Technology Ltd) and incubated at 4°C overnight. Lung leukocytes or splenocytes were stimulated with SARS-CoV-2 S and nucleocapsid (N) peptide pools (2 μg/mL, Miltenyi Biotec) for 36 h at 37°C. Cells were stimulated with anti-CD3 (1 μg/mL, e-Biosciences) or medium alone were used as positive and negative controls. This was followed by incubation with biotin-conjugated anti-IFN-γ at 1:100 dilution (Cellular Technology Ltd) for 2 h at room temperature, followed by incubation with alkaline phosphatase-conjugated streptavidin for 30 min. The plates were washed and scanned using an ImmunoSpot 6.0 analyzer and analyzed by ImmunoSpot software to determine the spot-forming cells (SFC) per 10^6^ lung leukocytes.

### Energy metabolism profile of lung γδ T cells

Metabolism profile analysis was performed as described previously with some modifications (39, 40). Briefly, 1 × 10^6^ lung leucocytes of mock or RSV-infected mice were stimulated with PMA (Sigma, 50 ng/mL) and Ionomycin (Sigma, 500 ng/mL) for 4 h. Cells were then either left untreated (control, Co) or treated with 2-deoxy-d-glucose (DG) (100 mM), oligomycin (O) (10 μM), and a combination of 2-DG and oligomycin (DGO) (100 mM and 10 μM) for 30 min. Following the addition of puromycin (10 μg/mL), the cells were incubated for an additional 45 min, and the cells were subsequently harvested and washed in cold FACS buffer before being stained with antibodies for TCRγδ and CD3 (ThermoFisher) for 20 min in ice. Cells were washed, fixed in 2% paraformaldehyde, and permeabilized with 0.5% saponin before adding mouse anti-puromycin-PE antibody (BioLegend, USA). Samples were acquired by a C6 Flow Cytometer instrument. Dead cells were excluded based on forward and side light scatter. Data were analyzed with a CFlow Plus Flow Cytometer (BD Biosciences). Based on puromycin mean fluorescence intensity (MFI) on gated γδ T cells, several parameters of the energetic metabolism profile metabolic, including glucose dependence, mitochondrial dependence, glycolytic capacity, and fatty acid and amino acid oxidation (FaaO) capacity, were calculated as described previously (39).

### RNA-seq analysis

RNA was extracted from lung tissues as described above and 2 µg of RNA was used for RNA-seq analysis. RNA samples quality was assessed using Agilent Bioanalyzer RNA Nano Chips (Agilent, Santa Clara, CA). RNAseq libraries were then prepared using NEBNext rRNA depletion kit v2 (Cat# 7400) and Ultra II Directional RNA library preparation kit (Cat# 7760) (NEB, Ipswich, MA) following manufacturer’s recommended procedure. The resulting libraries were run on Agilent Bioanalyzer High Sensitivity DNA Chips (Agilent, Santa Clara, CA) for size and quantified using qPCR. Sequencing was carried out on Element Biosciences Aviti sequencer (Element Biosciences, San Diego, CA) using paired end 75bp parameter to a sequencing depth of above 50 million paired reads per sample (57 million to 93 million). The reads were quality filtered and trimmed for adapter sequence using Trimmomatic-0.39 (41) and aligned to mouse GRCm39 reference genome using STAR 2.7.11a (42). Differential expression was performed using Bioconductor DESeq2 package (43) and GO enrichment analysis were performed using Bioconductor clusterProfiler package (44). GSEA analysis was performed using GSEA version 3.0 (45).

### Statistical analysis

Survival curve comparison was performed using GraphPad Prism software 9.4.1, which uses the log-rank test. Values for weight changes, viral load, cytokine production, antibody titers, and T cell response experiments were compared using Prism software statistical analysis and were presented as means ± SEM. *P* values of these experiments were calculated with a non-paired Student’s *t*-test, one-way ANOVA, or two-way ANOVA with Turkey’s multiple comparison tests.

## Data Availability

The RNA-seq data in this study were deposited in NCBI’s Gene Expression Omnibus under GEO Series accession number GSE300910.

## SUPPLEMENTAL MATERIAL

Figure S1 jvi.01658-25-s0001.tif Weight loss after RSV infection in K18-hACE2 mice.

Figure S2 jvi.01658-25-s0002.tif RNAseq analysis of lung samples of RSV-infected mice.

Figure S3 jvi.01658-25-s0003.tif RNAseq analysis of lung samples of SARS-CoV-2-infected mice with prior RSV infection.

Figure S4 jvi.01658-25-s0004.tif SARS-CoV-2 specific antibody responses in RSV-infected mice.

Figure S5 jvi.01658-25-s0005.tif RSV-induced gamma delta T cell responses contribute to heterologous protection against subsequent SARS-CoV-2 challenge.

Supplemental text jvi.01658-25-s0006.docx Supplemental methods and legends for Fig. S1 to S5.

Table S1 jvi.01658-25-s0007.xlsx Top 100 differentially expressed genes.

ASM does not own the copyrights to Supplemental Material that may be linked to, or accessed through, an article. The authors have granted ASM a non-exclusive, world-wide license to publish the Supplemental Material files. Please contact the corresponding author directly for reuse.
