# Supplementary figures and images for: Respiratory syncytial virus infection induces heterologous protection against SARS-CoV-2 through γδ T cell-mediated trained immunity and the activation of SARS-CoV-2–reactive mucosal T cells
Source: J Virol. 2026 Mar 18;100(4):e01658-25. doi: 10.1128/jvi.01658-25 (PMC13002117; doi:10.1128/jvi.01658-25)

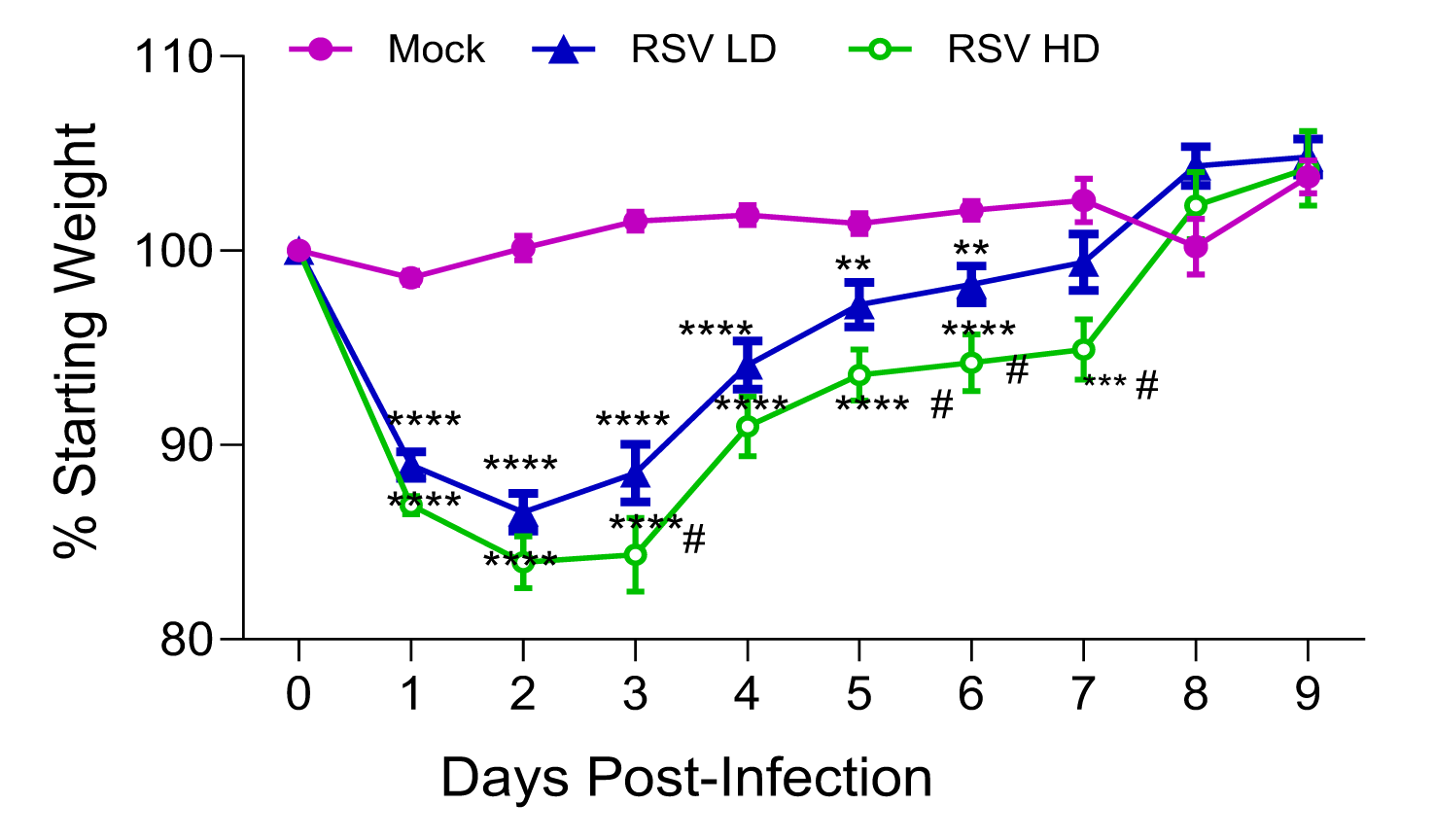

Supplement: Figure S1 — Weight loss after RSV infection in K18-hACE2 mice. [file jvi.01658-25-s0001.tif]

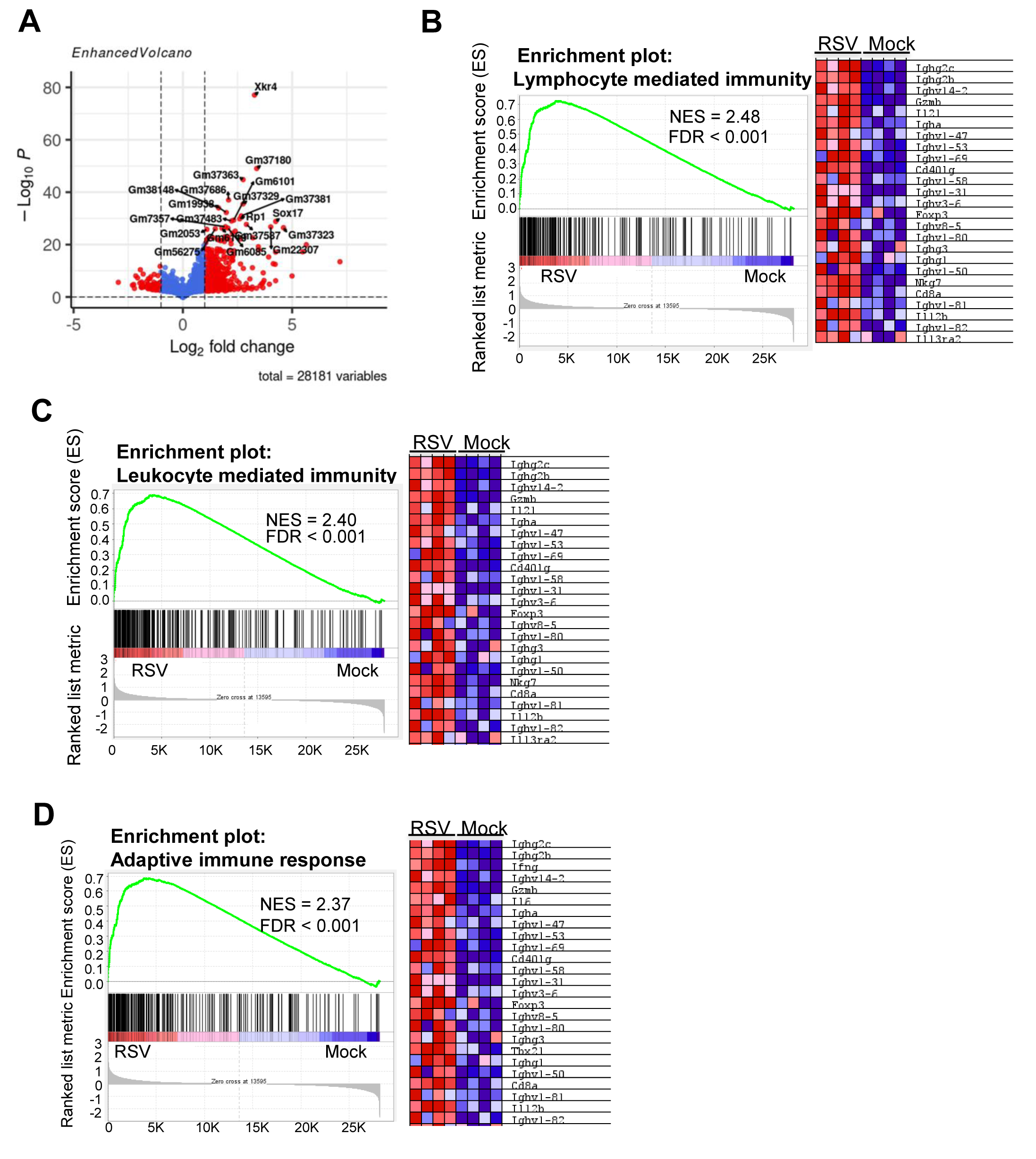

Supplement: Figure S2 — RNAseq analysis of lung samples of RSV-infected mice. [file jvi.01658-25-s0002.tif]

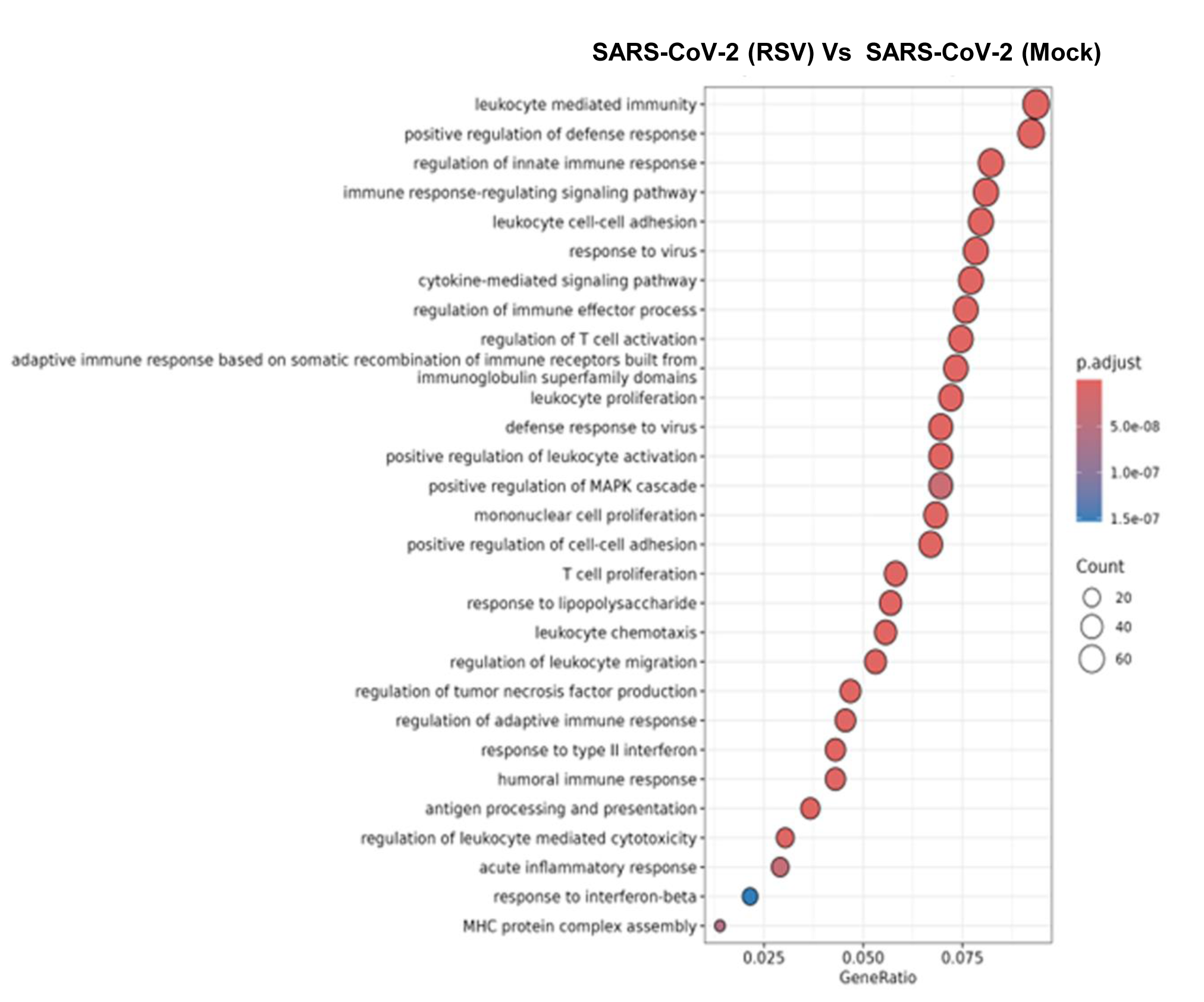

Supplement: Figure S3 — RNAseq analysis of lung samples of SARS-CoV-2-infected mice with prior RSV infection. [file jvi.01658-25-s0003.tif]

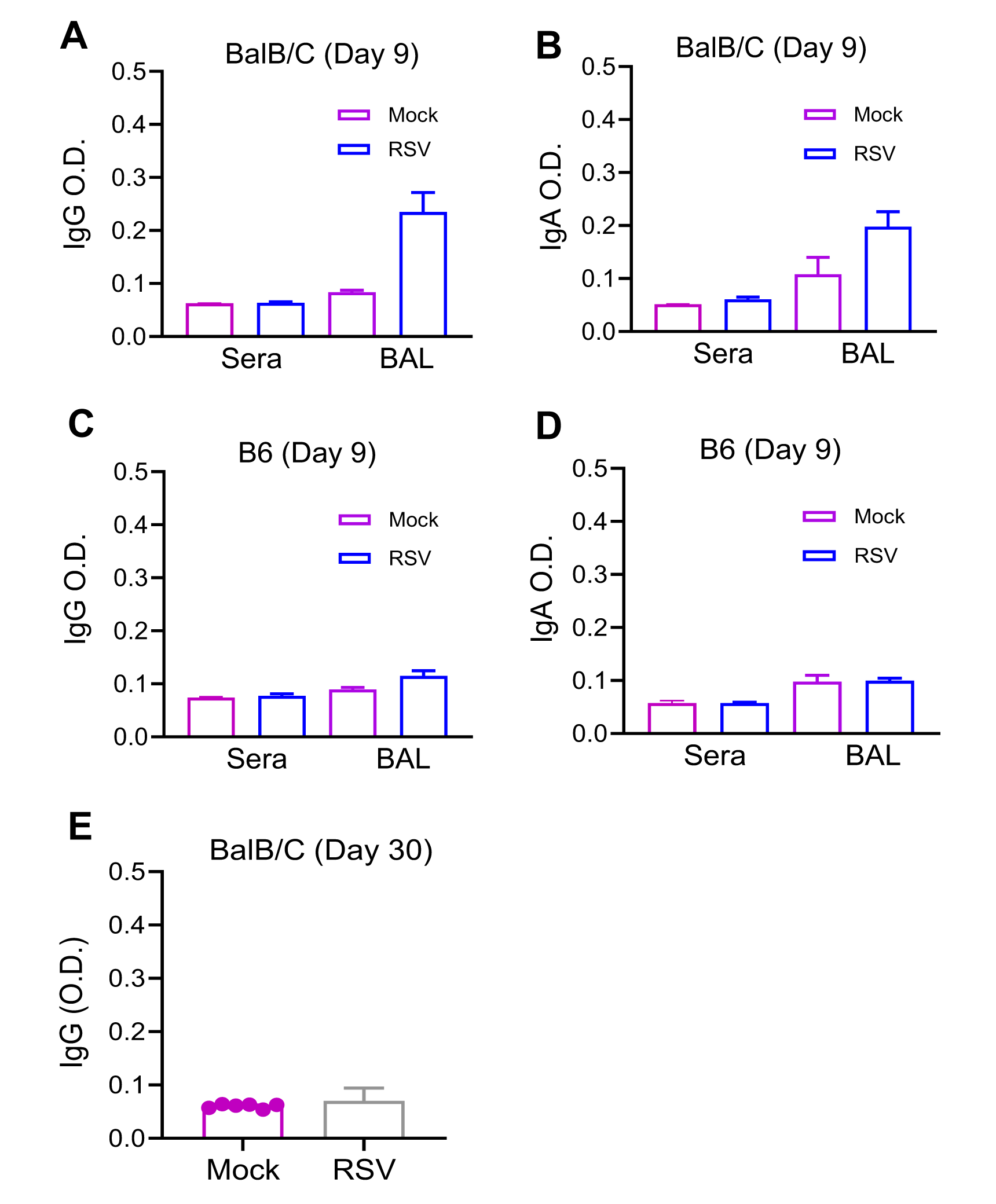

Supplement: Figure S4 — SARS-CoV-2 specific antibody responses in RSV-infected mice. [file jvi.01658-25-s0004.tif]

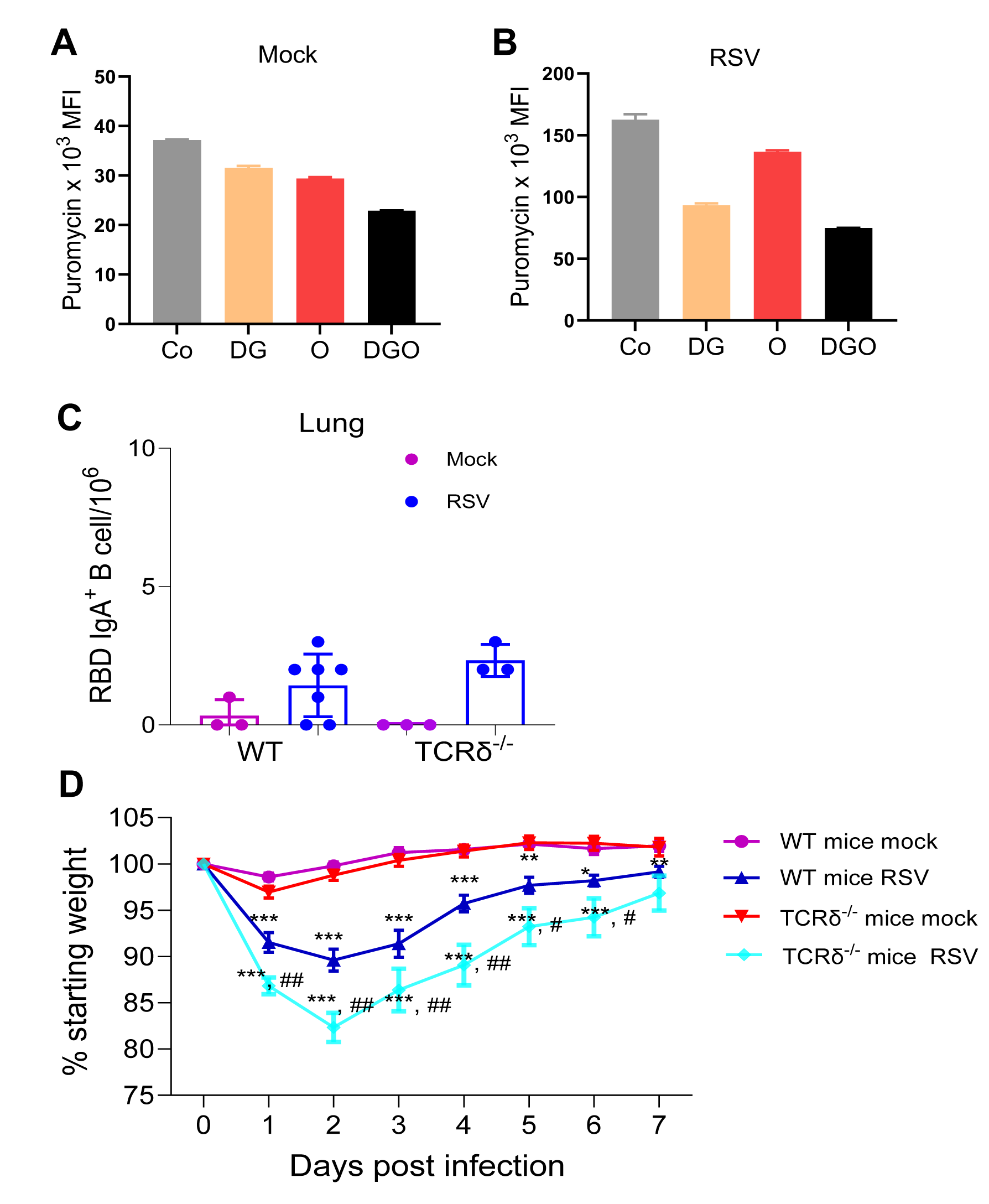

Supplement: Figure S5 — RSV-induced gamma delta T cell responses contribute to heterologous protection against subsequent SARS-CoV-2 challenge. [file jvi.01658-25-s0005.tif]
