# Supplementary material for: Respiratory syncytial virus infection induces heterologous protection against SARS-CoV-2 through γδ T cell-mediated trained immunity and the activation of SARS-CoV-2–reactive mucosal T cells
Source: J Virol. 2026 Mar 18;100(4):e01658-25. doi: 10.1128/jvi.01658-25 (PMC13002117; doi:10.1128/jvi.01658-25)
Supplement: Supplemental text — Supplemental methods and legends for Fig. S1 to S5. [file jvi.01658-25-s0006.docx]

**Supplementary Methods:**

**B cell ELISPOT**

Millipore ELISPOT plates (Millipore Ltd, Darmstadt, Germany) were coated with 100 µl of 15 µg/ml rSARS-CoV-2 RBD protein (RayBiotech). To detect total IgA^+^-expressing B cells, the wells were coated with 100 µl of anti-mouse IgA capture Ab 15 µg/ml, (Mabtech In). Cells were added in duplicate wells to assess total IgA ASCs or SARS-CoV-2 specific B cells. The plates were incubated overnight at 37˚C, followed by incubation with biotin-conjugated anti-mouse IgA (0.5 µg/ml, 3865-6, Mabtech In) for 2 h at room temperature, then 100 µL/well streptavidin-ALP (1:1000) was added for 1 h. Plates were developed with BCIP/NBT-Plus substrate until distinct spots emerge, washed with tap water, and scanned using an ImmunoSpot 6.0 analyzer and analyzed by ImmunoSpot software (Cellular Technology Ltd).

**Supplementary Figure Legends**

**Supplementary Figure 1:** **Weight loss after RSV infection in K18-hACE2 mice.** K18-hACE2 mice were infected with a low (LD) or high dose (HD) of RSV or mock. Mice were monitored daily for weight loss. Weight loss is indicated by percentage using the weight on the day of infection as 100%. *****P* < 0.0001, ****P* < 0.001, or ***P* < 0.01, LD (n= 23) or HD (n =10) group compared to mock- infected mice (n = 18). ^#^*P* < 0.05 compared to LD group.

**Supplementary Figure 2:** **RNAseq analysis of lung samples of RSV-infected mice.** B6 mice were infected i.n. with RSV or mock. Lung tissues were collected at day 9 post RSV or mock infection. Lung tissue RNA was used for RNAseq analysis. **A.** Volcano plot of RSV vs. Mock groups. Color dots represent genes that meet different criteria of analysis. Gray color:  No significance (does not meet either significance *P* value or foldchange cut off);  Green dots: Meeting only foldchange cutoff; Blue dots: meeting *P* value cutoff (multi-testing adjusted *P* < 0.1);  Red dots: meeting both fold change and *P* value cutoff. **B-D.** GSEA enrichment plots for immune-related pathways in RSV vs. mock group. Enrichment plots show upregulation of (**B**) lymphocyte-mediated immunity, (**C**) leukocyte-mediated immunity, and (**D**) adaptive immune response pathways in RSV-infected samples compared to mock. Normalized enrichment scores (NES) and false discovery rates (FDR) are indicated for each pathway. Heatmaps depict the relative expression levels of genes within (**B**) lymphocyte-mediated immunity, (**C**) leukocyte-mediated immunity, and (**D**) adaptive immune response pathways. Each row represents a gene, and each column represents a sample. Red indicates higher expression; blue indicates lower expression.

**Supplementary Figure 3:** **RNAseq analysis of lung samples of SARS-CoV-2- infected mice with prior RSV infection.** B6 mice were infected i.n. with RSV or mock, and on day 9 post infection, mice were challenged with SARS-CoV-2 CMA4. Lung tissues were collected at day 2 post SARS-CoV-2 challenge in mice with prior RSV or mock infection. Lung tissue RNA was used for RNAseq analysis. A simplified GO enrichment dot plot showing the most significant signaling pathways induced by SARS-CoV-2 challenge with prior RSV infection compared to SARS-CoV-2 infected mice with prior mock infection.

**Supplementary Figure 4:** **SARS-CoV-2 specific antibody responses in RSV-infected mice.** BALB/c (**A-B** & **E**) or B6 (**C-D**) mice were infected i.n. with RSV or mock. At day 9 (**A-D**) or day 30 (**E**) post infection, sera and BAL were collected. Results of IgG and IgA ELISA using SARS-CoV-2 recombinant RBD protein antigen were presented.

**Supplementary Figure 5:** **RSV-induced** **γδ T cell responses contribute to heterologous protection against subsequent SARS-CoV-2 challenge.**  **A-B**. Metabolic analysis by a modified SCENITH. Lung leukocytes were isolated from mock (**A**) or RSV (**B**)infected BALB/c mice at day 9 pi. Cells were gated on CD3^+^TCRγδ^+^ cells for puromycin incorporation, Data are presentive of three similar experiments. **C.** SARS-CoV-2 specific memory B cell (MBC) responses by ELISPOT analysis at day 9 post RSV infection. Frequencies of RBD specific ASCs per 10^6^ input cells in MBC cultures from the subject. **D.** WT B6 and TCRδ^-/-^ mice were infected i.n. with 5 x10^6^ PFU RSV A2 or mock. Mice were monitored daily for weight loss. Weight loss is indicated by percentage using the weight on the day of infection as 100%. ****P* < 0.001, ***P* < 0.01, or **P* < 0.05 WT mice RSV (n= 23) or TCRδ^-/-^ mice RSV (n =19) groups compared to WT mice mock (n =16) or TCRδ^-/-^ mice mock groups (n= 13) respectively. ^##^*P* < 0.01, or ^#^*P* < 0.05, WT mice RSV (n= 23) compared to TCRδ^-/-^ mice RSV (n= 19).
